# Symptom dimensions are associated with reward processing in unmedicated persons at risk for psychosis

**DOI:** 10.3389/fnbeh.2014.00382

**Published:** 2014-11-18

**Authors:** Diana Wotruba, Karsten Heekeren, Lars Michels, Roman Buechler, Joe J. Simon, Anastasia Theodoridou, Spyros Kollias, Wulf Rössler, Stefan Kaiser

**Affiliations:** ^1^University Hospital of Psychiatry Zurich, the Zurich Program for Sustainable Development of Mental Health Services (ZInEP)Zurich, Switzerland; ^2^Department of Neuroradiology, University Hospital of ZurichZurich, Switzerland; ^3^Collegium Helveticum, A Joint Research Institute between the University of Zurich and the Swiss Federal Institute of Technology ZurichZurich, Switzerland; ^4^Department of Psychiatry, Psychotherapy and Psychosomatics, University Hospital of PsychiatryZurich, Switzerland; ^5^Center for MR Research, University Children’s Hospital ZurichZurich, Switzerland; ^6^Department of General Internal Medicine and Psychosomatics, Centre for Psychosocial Medicine, University Hospital HeidelbergHeidelberg, Germany; ^7^Laboratory of Neuroscience (LIM-27), Institute of Psychiatry, University of Sao PauloSao Paulo, Brazil; ^8^Center for Integrative Human Physiology, University of ZurichZurich, Switzerland

**Keywords:** reward, salience processing, psychosis, ventral striatum, anterior insula, dopamine, at-risk mental state, functional magnetic resonance imaging (fMRI)

## Abstract

There is growing evidence that reward processing is disturbed in schizophrenia. However, it is uncertain whether this dysfunction predates or is secondary to the onset of psychosis. Studying 21 unmedicated persons at risk for psychosis plus 24 healthy controls (HCs) we used a incentive delay paradigm with monetary rewards during functional magnetic resonance imaging. During processing of reward information, at-risk individuals performed similarly well to controls and recruited the same brain areas. However, while anticipating rewards, the high-risk sample exhibited additional activation in the posterior cingulate cortex, and the medio- and superior frontal gyrus, whereas no significant group differences were found after rewards were administered. Importantly, symptom dimensions were differentially associated with anticipation and outcome of the reward. Positive symptoms were correlated with the anticipation signal in the ventral striatum (VS) and the right anterior insula (rAI). Negative symptoms were inversely linked to outcome-related signal within the VS, and depressive symptoms to outcome-related signal within the medial orbitofrontal cortex (mOFC). Our findings provide evidence for a reward-associated dysregulation that can be compensated by recruitment of additional prefrontal areas. We propose that stronger activations within VS and rAI when anticipating a reward reflect abnormal processing of potential future rewards. Moreover, according to the aberrant salience theory of psychosis, this may predispose a person to positive symptoms. Additionally, we report evidence that negative and depressive symptoms are differentially associated with the receipt of a reward, which might demonstrate a broader vulnerability to motivational and affective symptoms in persons at-risk for psychosis.

## Introduction

Subcortical dopamine dysregulation is a cornerstone in our understanding of schizophrenia (Howes and Kapur, 2009). There is general agreement on the central role of dopamine in mediating mesostriatal neural activity involved in reward processing, specifically in encoding motivational value and salience (Bromberg-Martin et al., 2010). Accumulating evidence suggests dysregulated dopaminergic transmission as a possible mediator for disturbances associated with altered processing of reward, incentive salience and learning in schizophrenia (Ziauddeen and Murray, 2010).

Both the anticipation and receipt of rewards have distinct neural correlates (Knutson et al., 2001b; Dillon et al., 2008; Berridge et al., 2009). The anticipatory phase involves activation in the ventral striatum (VS), encompassing the nucleus accumbens (NAcc; Knutson et al., 2001b; Schott et al., 2008) and the anterior insula (Volz et al., 2004; Knutson and Greer, 2008; Krebs et al., 2012). This anticipatory signal has been proposed to code the expected value of the predicted reward probability distribution rather than reward prediction error *per se* (Schultz, 2010). It is hypothesized that chaotic firing of dopaminergic neurons projecting to those regions mediates inadequate attribution of salience to irrelevant events, which might contribute to the formation of positive psychotic symptoms (Kapur et al., 2005; Palaniyappan and Liddle, 2012). Nielsen et al. (2012) whose study draws upon the concept of anticipation of reward being associated with salience processing, found a significant correlation between striatal activation during this stage to positive symptoms, agreeing with the aberrant salience theory.

During reward feedback, VS activation reflects prediction error in response to unexpected rewards (Schultz, 2002) while activity in the ventromedial/medial orbitofrontal cortex (mOFC) signals the updating of reward value (Grabenhorst and Rolls, 2011) and hedonic experience (Kringelbach, 2005). Accordingly, a deficit in the processing of reward receipt on both levels has been associated with anhedonia and depression, although the findings are more consistent for the VS than for the mOFC (McCabe et al., 2009; Pizzagalli et al., 2009; Simon et al., 2010a; Gradin et al., 2011). Dysfunctional activation during both anticipation and outcome in striatal and cortical regions has been associated with negative symptoms (Juckel et al., 2006; Simon et al., 2010a; Waltz et al., 2011). Some groups including our own have suggested that a higher specificity can be reached by investigating subdimensions of negative symptoms, which was not feasible in the context of this high-risk study.

There is consistent evidence that reward processing and associated cortico-striatal interactions are perturbed in schizo­phrenia (Heinz and Schlagenhauf, 2010; Simon et al., 2010b; Ziauddeen and Murray, 2010). Attenuated striatal responses during the anticipation of rewards have been primarily observed in unmedicated patients with schizophrenia (Juckel et al., 2006; Schlagenhauf et al., 2009), although medicated patients with more severe negative symptoms also seem to show a reduced signal (Simon et al., 2010a; Waltz et al., 2011). However, it is uncertain whether dysregulations of the reward system predate or follow the development of psychosis. Examining reward processing in at-risk individuals may provide further insight into illness susceptibility and its underlying pathophysiological mechanisms. Results from recent studies with positron emission tomography (PET) have suggested that dopaminergic dysregulation begins prior to the first psychotic episode, and importantly appears predictive of conversion to psychotic illness (Howes et al., 2009, 2011). Furthermore, motivational salience processing and associated responses in the VS (Roiser et al., 2013), as well as reduced activation during loss-avoidance anticipation in pre-psychotic individuals has been observed (Juckel et al., 2012).

Therefore, our goal was to explore functional brain correlates during both anticipation and receipt of rewards and to evaluate their association with symptoms in unmedicated persons at risk for psychosis. We compared the neural activation of HCs with an unmedicated at-risk group by administering a modified version of the monetary incentive delay task (Knutson et al., 2001a; Abler et al., 2005; Simon et al., 2010b). Regarding brain-symptom relationships the previous work cited above provides some evidence for differential associations between symptoms reward anticipation and outcome in patients with schizophrenia, although the findings are heterogeneous. Thus, we tested the following hypotheses: (1) positive symptoms are associated with activation of the VS and the anterior insula during reward anticipation, (2) negative symptoms are associated with reduced VS activation during reward anticipation; and (3) depressive symptoms are associated with reduced VS and mOFC activation during processing of rewarding outcomes.

## Materials and methods

### Participants

This project consisted of 21 medication-free participants at risk for psychosis (Risk) and 24 healthy controls (HC). Participants were recruited in the region of Zurich, Switzerland, within the frame of a larger study on early detection of psychosis,^1^ which was approved by the cantonal Ethic Commission Zurich (E-63/2009) and complies with the Declaration of Helsinki.

For the present study, psychopathology (i.e., positive and negative symptoms) was rated with the Structured Interview for Psychosis-Risk Syndrome (SIPS; Miller et al., 2003), the Schizophrenia Proneness Instrument (SPI-A; Schultze-Lutter et al., 2007), and the Calgary Depression Scale for Schizophrenia (CDSS; Addington et al., 1993). All participants in the Risk group fulfilled inclusion criterion for high-risk status as assessed by the SPI-A, which was met when at least one cognitive-perceptive basic symptom or at least two cognitive disturbances were reported. Six individuals in the Risk group reported at least one attenuated psychotic symptom or brief, limited intermittent psychotic symptom as assessed by the SIPS, and thus fulfilled additionally the criterion for UHR status. Imaging of the participants was conducted immediately after entry into the ZInEP study before onset of any treatment.

Persons in the HC group were screened with the Mini-International Neuropsychiatric Interview (Sheehan et al., 1998) to ensure that none had any history of psychiatric illness. Individuals in the Risk and HC groups did not differ significantly in terms of age, gender, handedness (assessed with the Edinburgh Handedness Inventory; Oldfield, 1971), and intelligence (estimated by using tests measuring both verbal (Mehrfachwahl-Wortschatz-Intelligenz Test; MWT-B; Lehrl, 2005) and nonverbal intelligence (Leistungsprüfsystem; LPS-3; Horn, 1983; Table 1). Exclusion criteria for both groups were age under 16 or over 35 years, contraindications against MRI, neurological illness, and substance abuse.

**Table 1:** **Demographic characteristics and symptom ratings**.

	HC	Risk	Statistical evaluation
N	24	21	
Gender (f:m)	11:13	6:15	χ² = 1.42, n.s.*
Handedness (r:l:b)	21:2:1	19:1:1	χ² = 0.23, n.s.*
Age (years)	23.3 ± 5.0	25.1 ± 5.6	*t* = −1.8, *n.s*†
Estimated intelligence	115.8 ± 14.4	111.6 ± 14.4	*t* = 1.0, *n.s*†
SIPS:			
- Positive	—	6.5 ± 3.9	—
- Negative	—	9.8 ± 5.8	—
- General	—	6.6 ± 3.0	—
- Disorganization	—	2.5 ± 2.2	—
GAF	—	58.2 ± 19.0	—
CDSS	—	6.5 ± 2.7	—

HC, healthy controls; Risk, subjects at risk for psychosis; r:l:b, right, left, both/bimanual; SIPS, symptoms according to Structured Interview for Prodromal Syndromes; GAF, Global Assessment of Functioning Scale Mean; CDSS, Calgary Depression Scale for Schizophrenia. *Pearson’s chi-square test; ^†^2-sample t-test; n.s., not significant (p > 0.05), ± SD where appropriate. Estimated intelligence was based upon mean scores from evaluations of verbal (MWT-B; Lehrl, 2005) and nonverbal (LPS-3; Horn, 1983) skills.

### Experimental design and task

**Figure 1:**
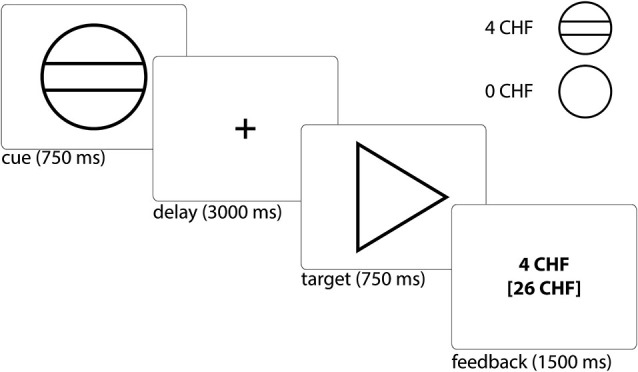
**Monetary incentive delay task: Example trial and cues representing possible reward outcomes**. Participants first saw a cue stipulating with an unpredictable probability the amount of money (4 CHF or 0 CHF) they could win, if they reacted correctly within 750 ms during the ensuing discrimination task, which involved pressing either a left or right button depending upon the direction of a triangle after an anticipation period (variable delay: 2500–3500 ms, mean of 3000 ms). Immediately after target presentation, subjects were informed about the amount of money they had won during this trial and their cumulative total win so far (feedback) for a total of 1500 ms (Abler et al., 2005). The jittered inter-trial interval (ITI) was between 1000 and 8000 ms with a mean of 4000 ms. Trial types were randomly ordered.

We used a modified version of the monetary incentive delay task (Figure 1), which has been proven to be a useful probe of neural responses during reward anticipation and receipt. To minimize learning effects during the fMRI, the MID-task was explained carefully by showing each cue and its meaning to the subjects. Participants had to perform a practice version of the task containing 10 trials, for which they did not receive payment. They were also shown the money they could earn by performing the task successfully in the scanner. During functional scan acquisition the test subjects engaged in one session with 50 trials. Two levels of reward were possible: 0 Swiss Francs (CHF) or 4 CHF, with a maximum overall win of 60 CHF. A steady rate of reward vs. non-reward across all participants was accomplished by applying a probabilistic pattern, which entailed no reward being paid in 10 pre-defined trials (out of the 25 trials with a potential reward).

The cue was presented for 750 ms and was followed by a variable delay of 2500–3500 ms (mean 3000 ms). A fixed response time window of 750 ms was chosen in order to ensure low task difficulty with a very high success rate, i.e., the rate of reward vs. non-reward depended little on the subjects’ performance. This procedure was chosen to (i) avoid confounding effects of psychomotor slowing and cognitive impairment (Demjaha et al., 2012; Fusar-Poli et al., 2012; Lin et al., 2012); and (ii) to avoid creating a stressful task environment. Finally, the participant received feedback on the money won in the current trial and the total amount of money won, which was presented for 1500 ms. In order to be sensitive to differences between trials close together in time we used a variable inter-trial interval (ITI), chosen from a Gaussian distribution with range of 1000 to 8000 ms, step of 500 ms and mean of 4000 ms. We measured reaction times (RTs) to cues with potential rewards in order to measure motivation.

### Imaging data acquisition

Functional and structural MRI was performed at the Psychiatric Hospital, University of Zurich, Switzerland, using a Philips Achieva TX 3-T whole-body MR unit with an eight-channel head coil. Three-dimensional T1-weighted anatomical images were acquired (160 slices; repetition time (TR) = 1900 ms; TE = 2.2 ms; inversion or echo time (TE) = 900 ms; flip angle *θ* = 78°; spatial resolution, 1 × 1 × 1 mm). Functional scans included a T2^*^-weighted echoplanar imaging sequence (265 volumes; TR = 2000 ms; TE = 30 ms; 32 contiguous, inter-leaved slices; spatial resolution, 3 × 3 × 3 mm; *θ* = 80°). To minimize susceptibility artifacts in the mOFC, we placed the contiguous axial slices at a 20^°^ angle relative to the anterior-posterior commissural plane. Participants viewed visual stimuli with LCD video goggles (Resonance Technologies). Responses were recorded with a Lumitouch response box (Photon Technologies).

### fMRI data analysis

Our fMRI data were analyzed using SPM8^2^. The pre-processing steps included realignment, in which fMRI-time series were rigidly registered to a reference image in order to correct for motion artifacts, slice-timing correction, co-registration to a structural T1 scan, spatial normalization to MNI space, and spatial smoothing (8-mm Gaussian kernel). Three of the participants were excluded due to excessive head motion (i.e., linear shift >2 mm, rotation >1°).

A general linear model was constructed for statistical analysis (Friston et al., 1994). Regressors for the two phases of anticipation (expectation of 4 CHF or 0 CHF) and three phases of outcome (receipt of 4 CHF, omission of 4 CHF, or receipt of 0 CHF/neutral outcome) were modeled separately as explanatory variables convolved with the canonical HRF. The six realignment parameters were included together with the onsets of targets and error-trials as regressors of no interest. To examine the anticipation of reward, we contrasted “*anticipation of *4 *CHF* vs. *anticipation of* 0 *CHF*”. The reward outcome was modeled by contrasting “*receipt of reward* vs. *omission of reward*”, i.e., we contrasted outcome regressors for which the preceding anticipation was the same (anticipation of 4 CHF). Thus, although the timing of the task did not allow for a definite separation of the trial phases within the temporal resolution of fMRI, our selection of contrasts nevertheless allowed comparisons between the trial-types of interest.

The individual contrast images were then subjected to a second-level random effects analysis. Within-group activation was compared using a one-sample *t*-test. The initial threshold for group-level maps was *p* < 0.001 (uncorrected). Given our strong *a priori* hypothesis regarding involvement of the VS and rAI in the processing of anticipation rewards and mOFC in the feedback of rewards, we employed family-wise error level correction adjusted for small volume (*P*_SVC_) across each of our independently derived regions of interest (ROIs) at the voxel level. For the VS, we used anatomical voxel masks for the left and right hemispheres, as retrieved from a publication-based probabilistic MNI-atlas (Nielsen and Hansen, 2002). This method has been used in previous reward-related fMRI studies (Juckel et al., 2006; Schlagenhauf et al., 2009; Simon et al., 2010a). For the mOFC, we used a functional ROI based on an earlier reward-related fMRI investigation with healthy participants using the same paradigm (Simon et al., 2010b). Finally, we selected a rAI ROI, because aberrant activation of this brain region has been previously reported in a high-risk sample (Wotruba et al., 2014) and has been suggested to be relevant for the pathomechanisms underlying the development of positive symptoms (Palaniyappan and Liddle, 2012). Importantly, rAI activation has also been shown during anticipation of a reward (Knutson and Greer, 2008; Krebs et al., 2012). We selected a spherical ROI centered on MNI coordinates (*x* = 38, *y* = 22, *z* = −10; 10 mm radius) (Seeley et al., 2007; Wotruba et al., 2014). The corresponding ROI’s are depicted in Figures 3A, 4A.

**Figure 2:**
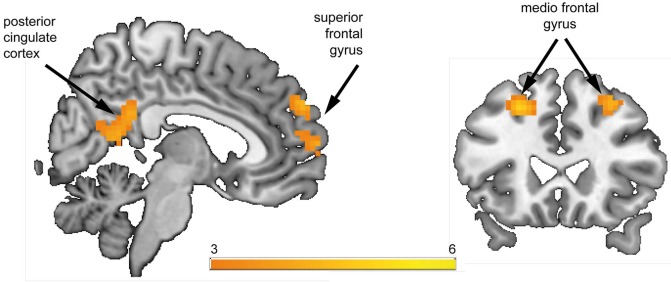
**Whole-brain group comparison of the contrast reward anticipation vs. no reward anticipation**. Subjects at risk for psychosis showed significantly stronger hemodynamic response compared to healthy controls in the posterior cingulate cortex, superior frontal gyrus, and bilateral medio frontal gyrus (corresponding *t*-values are represented in orange/yellow).

**Figure 3:**
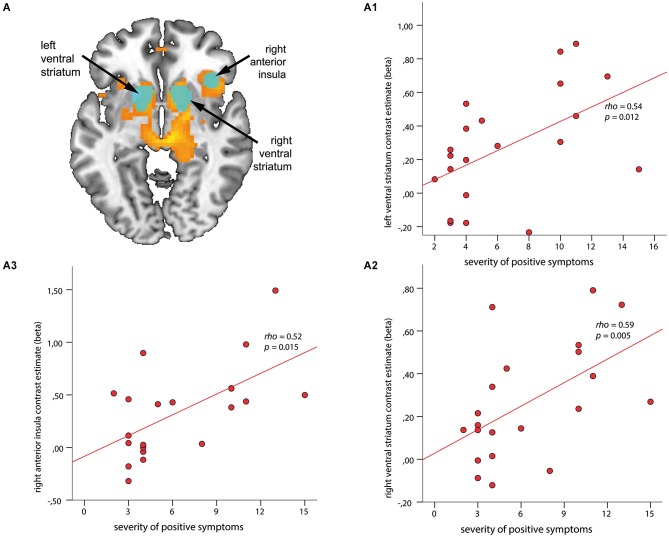
**Associations between regions of interest (ROI) and severity of positive symptoms during anticipation of reward**. ROIs (depicted in cyan) are overlaid on within-group t-maps for subjects at risk for psychosis **(A)** for the contrast reward anticipation vs. no reward anticipation (shown in orange, both at a voxel-wise threshold *p* < 0.001, with an extent of 20 voxels). ROI-based analysis revealed significant association between contrast estimates of the left and right ventral striatum (VS) **(A1, A2)** and right anterior insula (rAI) **(A3)** with positive symptom scores (*ρ* > 0.52, *p* < 0.015).

**Figure 4:**
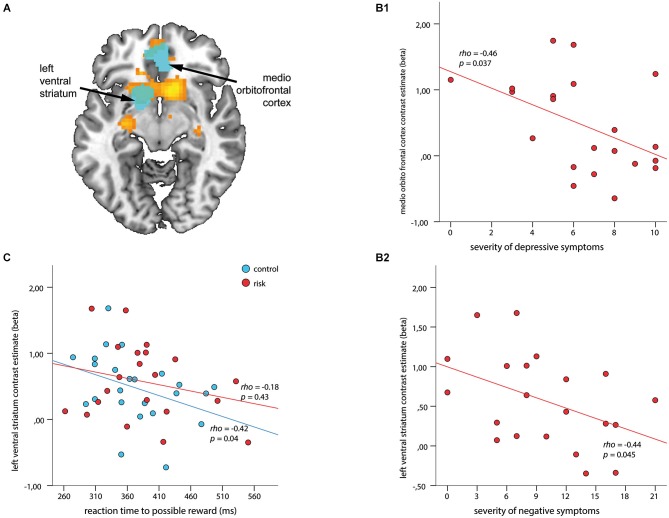
**Associations among regions of interest (ROIs), clinical symptoms, and reaction time (RT) to cues with possible reward during outcome**. ROIs (depicted in cyan) are overlaid on the within-group t-map for subjects at risk for psychosis **(A)** for the contrast receipt of reward vs. omission of reward (shown in orange, both at a voxel wise threshold of *p* < 0.001, with an extent of 20 voxels). ROI-based analysis revealed a negative association between contrast estimates within the medio orbitofrontal cortex and severity of depressive symptoms **(B1)**, and the left VS and severity of negative symptoms **(B2)** (*ρ* > −0.44, *p* < 0.045). **(C)** Signal in the left VS revealed a significant inverse association with RT in healthy controls (blue; *ρ* = −0.42, *p* = 0.04) but not for subjects at risk for psychosis (red; *ρ* = −0.18, *p* = 0.43).

Individual parameter estimates (beta-values) were extracted using the mean of the data, collapsed across all voxels within each ROI using the REX toolbox^3^, and were correlated to symptom scores (SIPS Negative, SIPS Positive, and CDSS) as well as with RT via Spearman’s correlation analysis. Significant results are reported at *p* < 0.05. No correction for multiple testing was applied to the correlational analyses.

In addition, we performed a whole-brain analysis using the aforementioned contrasts to identify group differences in brain areas outside the ROIs. The threshold was set to voxelwise *p* < 0.001 and 20 contiguous voxels, corresponding to a false-positive discovery rate of *p* < 0.05 across the whole brain as estimated by Monte Carlo simulation.

All raw data are available from the corresponding author on request.

## Results

### Behavioral results

The average error rate for all subjects was 2.1%. Participants were significantly faster in trials when 4 CHF was promised (mean 379.3 ms, SD 6.7) than when they expected no reward (mean 406.3 ms, SD 5.2; *t* = 3.3, *p* = 0.002). The groups did not differ significantly in either RTs (*p* > 0.5) or error rates (*p* > 0.2).

### Anticipation

#### Within group activations during reward anticipation

We first analyzed within group activations to anticipation of possible rewards (i.e., *anticipation of* 4 *CHF vs. anticipation of* 0 *CHF)* in each of our a priori defined regions of interest. Both HC and Risk groups displayed significant hemodynamic responses within the left VS (HC: *z* = 4.81, *P*_SVC_ < 0.000; Risk: *z* = 4.62, *P*_SVC_ = 0.004), right VS (HC: *z* = 5.19, *P*_SVC_ < 0.001; Risk: *z* = 4.79, *P*_SVC_ = 0.003), and rAI (HC: *z* = 4.31, *P*_SVC_ < 0.001; Risk: *z* = 3.48, *P*_SVC_ = 0.004). Only at-risk persons exhibited activation within the mOFC (*z* = 3.32, *P*_SVC_ = 0.03).

#### Between group comparisons during reward anticipation

In the a priori defined ROIs (VS, rAI, mOFC) no significant differences between HC and Risk groups were observed. We performed an exploratory whole brain analysis, which revealed significantly increased hemodynamic responses in the Risk vs. HC group within the following regions: posterior cingulate cortex [PCC; Brodmann Area (BA) 31; *x* = 3, *y* = −45, *z* = 27; cluster size = 123 voxels], superior frontal gyrus (SFG; BA 9; *x* = 9, *y* = 57, *z* = 30; cluster size = 41 voxels), bilateral medial frontal gyrus (MFG; BA 8; *x* = 30/−24, *y* = 24, *z* = 48/45; cluster size = 36/50 voxels) (Figure 2). Activations were not significantly increased in any brain region for the HC subjects relative to the Risk group.

#### Correlations between ROI activation and psychopathology

The ROI-based analysis revealed significant correlations between the SIPS positive symptom score and hemodynamic response in the left VS (*ρ* = 0.54, *p* = 0.012; Figure 3A1) and right VS (*ρ* = 0.59, *p* = 0.005; Figure 3A2), as well as in the rAI (*ρ* = 0.52, *p* = 0.015; Figure 3A3). No significant association was found between regional brain activation and negative or depressive symptoms during the phase of reward anticipation.

### Outcome

#### Within group activations during reward outcome

We first analyzed the contrast *receipt of reward* vs.* omission of reward* in each of our a priori defined regions of interest for each group separately. Both groups showed significant hemodynamic responses within the mOFC (HC: *z* = 4.93, *P*_SVC_ < 0.000; Risk: *z* = 3.33, *P*_SVC_ = 0.036), left VS (HC: *z* = 4.80, *P*_SVC_ < 0.000; Risk: *z* = 4.03, *P*_SVC_ = 0.002), and right VS (HC: *z* = 4.87, *P*_SVC_ < 0.000; Risk: *z* = 4.55, *P*_SVC_ < 0.000), but not within the rAI.

#### Between group comparison during reward outcome

No significant group differences were found within the a priori defined ROIs. An exploratory whole brain analysis did not reveal any additional regions with significant between group differences.

#### Correlations between ROI activation and psychopathology

The ROI based correlations for the contrast *receipt of reward* vs. *omission of reward* revealed negative correlations for depressive symptoms with contrast estimates within the mOFC (*ρ* = −0.46, *p* = 0.037; Figure 4B1), and for negative symptoms with the left VS (*ρ* = −0.44, *p* = 0.045; Figure 4B2). No significant association with positive symptoms could be observed. An additional correlation analysis revealed a significant inverse relationship between RT during the 4 CHF condition and the outcome signal in the left VS (*ρ* = −0.42, *p* = 0.04) for the HC group but not for the Risk group (Figure 4C).

## Discussion

In our study, unmedicated individuals at risk for psychosis showed similar error rates and RTs as HCs during a monetary incentive delay task. The task was intended to produce low error rates, which lead both groups to perform at ceiling. Both groups recruited similar brain areas when processing reward information. However, during the anticipation phase, those in the Risk group exhibited additional activation in the PCC, MFG, and SFG. During receipt of rewards, the two groups did not differ significantly. Importantly, the neural processing of anticipation and receipt of rewards was differentially related to symptom dimensions. Positive symptoms were associated with the processing of reward anticipation, while negative and depressive symptoms were related to the processing of a rewarding outcome.

The lack of a significant group difference in the VS during reward processing contrasts with earlier findings from unmedicated patients with schizophrenia (Juckel et al., 2006; Schlagenhauf et al., 2009; Esslinger et al., 2012), in which activation in the VS was diminished during the anticipation phase. This may have been due to variations in experimental designs, i.e., the only previous study employing this monetary incentive delay task in a (partially-medicated) high-risk sample (Juckel et al., 2012), found no group differences in the VS during reward anticipation.

During the anticipation period, higher activation in the SFG and MFG was observed in the Risk group. Therefore, the impending action might have required increased effort to maintain task performance, which led to increased frontal activation. Compensatory hyperactivation of these regions has repeatedly been reported in patients with schizophrenia (Potkin et al., 2009; Deserno et al., 2012). Noteworthy, recent findings (Guitart-Masip et al., 2011) show that anticipatory signals capture some aspects of response preparation, which, in turn, may be related to the frontal hyperactivation shown by subjects in the at-risk group. However, our task does not allow differentiating between response preparation and reward anticipation, which would require future studies. We also found significantly stronger activation in the PCC for the Risk group compared with HC. The PCC is a key node of the default mode network, which, in healthy individuals, activates during rest periods, but deactivates during goal-directed tasks (Fox et al., 2005). Therefore, similar to reports with schizophrenia (Whitfield-Gabrieli and Ford, 2012), our result indicates less task-dependent deactivation of the PCC in the risk state for psychosis.

A central finding of our study is that positive symptoms are correlated with VS and rAI activation during reward anticipation. Dysfunctional activation of VS and rAI has been associated with aberrant assignment of salience to otherwise irrelevant stimuli, which might be part of the neuropathophysiological mechanism leading to psychotic symptoms (Jensen et al., 2008; Palaniyappan and Liddle, 2012). Consistent with this, patients with higher positive symptom scores have been observed to elicit greater hemodynamic responses in the VS to neutral stimuli (Jensen et al., 2008; Romaniuk et al., 2010; Roiser et al., 2013). In contrast, our at-risk participants, with a higher degree of sub-clinical positive symptoms, displayed a stronger signal response to meaningful cues. Thus, individuals with potentially prodromal symptoms might be predisposed to over-attributing salience to *any* event, which might reflect a sign for aberrant salience signaling after the onset of overt psychosis. In contrast to our own results, recent reports with unmedicated schizophrenic patients have pointed to an *inverse* relationship between VS activation and positive symptoms (Esslinger et al., 2012). These different results could be due to the fact, that previous studies employed a salience contrast involving losses while our trials included only reward contrasts.

In addition, a recent report shows that first-degree relatives of patients with schizophrenia show a decrease in VS activation during reward anticipation, which is also influenced by a polymorphism in the neuregulin-1 gene (Grimm et al., 2014). The fact that at-risk participants in our study did not show reduced VS activation in association with reward anticipation might reflect our different method of identifying individuals with increased likelihood of developing psychotic illness (based on subclinical symptoms rather than genotypes).

We observed an inverse relationship between the severity of negative symptoms and VS activation during the receipt of reward. This finding was somewhat unexpected, because in patients with schizophrenia an association of ventral striatal hypoactivation and negative symptoms was mainly reported for the anticipation phase (Juckel et al., 2006; Simon et al., 2010a; Waltz et al., 2010). Nevertheless, dysfunctional outcome processing has also been suggested to be associated with negative symptoms (Waltz et al., 2013), although these findings relate to prefrontal cortical regions. In addition, stronger activation of the VS during the reward phase was associated with faster RTs in HC participants, but not in Risk participants. This implies a dysregulation of the VS in subjects at-risk for psychosis that might affect the positive impact of rewarding actions and, consequently, contribute to the development of negative symptoms.

Furthermore, individuals with higher scores for depressive symptoms exhibit less activation within the mOFC, a region involved in immediate and simple hedonic responses (Kringelbach, 2005). Thus, reduced coding of pleasurable experiences in the mOFC may contribute to the neurobiological origin of depressive symptoms in at-risk persons. In contrast to our previous study in patients with schizophrenia, no association between depressive symptoms and VS activation during outcome was observed (Simon et al., 2010a). For both negative and depressive symptoms, one might speculate that individuals with higher symptom scores show less differentiation between positive and negative outcomes due to unregulated dopamine firing (Schlagenhauf et al., 2009; Heinz and Schlagenhauf, 2010).

A possible shortcoming of the present study is the relatively small sample size. Another constraint is the cross-sectional design, which could limit the relevance of our results. In addition, the correlations between RT and clinical symptom scores with the hemodynamic response in our ROIs were not corrected for multiple comparisons, which warrant caution in interpreting these findings until independently replicated. In addition, we pooled data from participants fulfilling ultra-high-risk and basic symptom criteria. Therefore, this did not allow us to attribute our findings specifically to either of those types of symptoms. Finally, the relationship between salience, value and reward prediction signals is still a matter of intense debate (Morris et al., 2012; Kahnt and Tobler, 2013). Our task is limited in its capacity to specifically attribute activation during reward anticipation and outcome to one of these signals.

In summary, our results provide evidence for a dysregulation of reward-associated processing in subjects at risk for psychosis, which could be compensated by the recruitment of prefrontal regions. Importantly, higher activation in the striatal and insular regions when anticipating reward-relevant cues might reflect abnormal processing of potential rewarding outcomes. This in turn could lead to a higher risk for developing supra-threshold psychotic disorder, which is in line with the aberrant salience theory of psychosis. Finally, we showed that negative and depressive symptoms are differentially related to VS and mOFC during the receipt of reward. Such a relation may reflect a broader vulnerability for motivational and affective symptoms in at-risk persons.

## Funding

This work was supported by the Zurich Program for Sustainable Development of Mental Health Services (ZInEP).

## Conflict of interest statement

The authors declare that the research was conducted in the absence of any commercial or financial relationships that could be construed as a potential conflict of interest.

## Footnotes

^1^www.zinep.ch

^2^http://www.fil.ion.ucl.ac.uk/spm

^3^http://web.mit.edu/swg/software.htm
